# High Metabolic Function and Resilience of NKG2A-Educated NK Cells

**DOI:** 10.3389/fimmu.2020.559576

**Published:** 2020-09-30

**Authors:** Andrew J. Highton, Björn-Philipp Diercks, Franziska Möckl, Gloria Martrus, Jürgen Sauter, Alexander H. Schmidt, Madeleine J. Bunders, Christian Körner, Andreas H. Guse, Marcus Altfeld

**Affiliations:** ^1^Research Department Virus Immunology, Heinrich Pette Institute, Leibniz Institute for Experimental Virology, Hamburg, Germany; ^2^The Calcium Signaling Group, Department of Biochemistry and Molecular Cell Biology, University Medical Center Hamburg-Eppendorf, Hamburg, Germany; ^3^Deutsche Knochenmarkspenderdatei (DKMS), Tübingen, Germany

**Keywords:** immunometabolism, NK cell education, KIR, NKG2A, glycolysis, oxidative phoshorylation

## Abstract

Natural killer (NK) cells are an important component of the innate immune system for the control of intracellular pathogens and cancer cells. NK cells demonstrate heterogeneous expression of inhibitory surface receptors. Signaling through these various receptors during NK cell development promotes functionality, referred to as NK cell education. Here we investigated the impact of education on NK cell metabolism through functional assessment of critical metabolic pathways and calcium signaling. Educated NK cells had an increased uptake of the metabolic substrates 2-NBDG, a fluorescent glucose analog, and BODIPY FL C_16_, a fluorescent palmitate, compared to uneducated NK cells. Comparison of NK cells educated *via* KIRs or NKG2A showed that NKG2A-educated NK cells were the main contributor to these differences in uptake of metabolites, and that NKG2A-educated NK cells were functionally more resilient in response to metabolic blockade of oxidative phosphorylation. Furthermore, NKG2A-educated NK cells exhibited higher peak calcium concentration following stimulation, indicating stronger signaling events taking place in these educated NK cells. These results demonstrate that cellular metabolism plays an important role in the functional differences observed between educated and uneducated NK cells, and show that NKG2A-educated NK cells remain more functionally competent than KIR-educated NK cells when oxidative phosphorylation is restricted. Understanding metabolic programming during NK cell education may unveil future targets to manipulate NK cell function for use in clinical settings, such as cancer therapies.

## Introduction

Natural killer (NK) cells are an integral part of the innate immune system and contribute to the control of viral infections through direct cytotoxicity against infected cells and through the release of cytokines. The activation threshold of NK cells is variable depending upon their education state, which is determined during development by the surface expression of inhibitory NK cell receptors, including Killer-cell Immunoglobulin-like Receptors (KIRs) and NKG2A (1–3). KIR2DL1/2/3, KIR3DL1, and NKG2A are inhibitory receptors expressed by NK cells that recognize different HLA class I molecules as their ligands. KIR2DL1 specifically binds to HLA-C group 2 molecules while KIR2DL2 and KIR2DL3 predominantly recognize HLA-C group 1 molecules. KIR3DL1 binds the HLA-Bw4 epitope, found primarily on HLA-B allotypes, and NKG2A recognizes the non-classical HLA-E molecule (4–6). Together, these inhibitory receptors' binding specificities cover a large proportion of the expressed HLA class I repertoire. It has been demonstrated that NK cells that express inhibitory receptors with available cognate HLA ligands have a greater cytotoxic potential compared to those that do not express inhibitory receptors or, alternatively, those that lack cognate ligands for their receptors (1–3, 7, 8). The exact mechanisms guiding this education process are not fully understood, but it has been recently suggested that differences in secretory lysosome size and calcium signaling may play a role (9). Calcium flux is known to be important for the functional response of NK cells and may underlie some of the functional differences observed between educated and uneducated NK cells (10).

Metabolic processes occurring within an immune cell are critically involved in determining their function. This has been well-defined in T lymphocytes, where it has been observed that the more quiescent naïve and memory T cells preferentially use oxidative phosphorylation for energy requirements while effector T cells commonly rely upon glycolysis to support their rapid proliferation and differentiation (11). In resting NK cells, low levels of oxidative phosphorylation and glycolysis are maintained, however following cytokine stimulation both glycolysis and oxidative phosphorylation are upregulated, suggesting a role for both pathways in NK cell function (12). Glucose is an important metabolite which fuels both glycolysis and oxidative phosphorylation, and inhibition of glycolysis with 2-DG or culture in glucose-free conditions has been shown to reduce IFN-γ production by NK cells (13, 14). In mice, cytokine stimulation of NK cells with IL-2 and IL-12 has been shown to cause upregulation of aerobic glycolysis and activation of the citrate-malate shuttle *via* glucose-derived citrate, bypassing the citric acid cycle for oxidative phosphorylation (15). These and other results establish glucose as an important metabolite for NK cell function, and initial studies have indicated differences in glucose metabolism between educated and uneducated NK cells (16, 17). In addition to glucose, fatty acids are important substrates for oxidative phosphorylation and their usage can skew immune cells' functionality (18). At present, the effect of differential usage of glucose and fatty acid on the metabolism of NK cells and on the modulation of NK cell education and function are not known. Here, we assessed critical metabolic pathways in educated and uneducated NK cells, and observed that both glucose and fatty acid uptake were increased in educated compared to uneducated NK cells. Furthermore, NK cells educated *via* NKG2A had superior metabolic function and higher metabolic resilience compared to NK cells educated *via* KIRs, and also exhibited increased peak calcium signaling following activation, driving enhanced NK cell responses.

## Materials and Methods

### Donor Cohort

Peripheral blood samples were collected from healthy blood donors recruited at the University Medical Center Hamburg-Eppendorf, Hamburg, Germany. The donors provided written informed consent and studies were approved by the ethical committee of the Ärztekammer Hamburg (PV4780). Peripheral blood mononuclear cells (PBMCs) were isolated by density-gradient centrifugation before resuspension in RPMI 1640 (Thermo Fisher Scientific, Waltham, MA, USA) supplemented with 10% (v/v) heat-inactivated fetal bovine serum (FBS) (Biochrom, Berlin, Germany). KIR and HLA typing was carried out per donor as previously described (19, 20).

### Cell Lines

K562 cells (DSMZ, Germany) were used as targets for NK cell stimulation. Target cell lines were grown in RPMI, supplemented with 10% (v/v) heat-inactivated FBS.

### Flow Cytometry

PBMCs in suspension were incubated in PBS with 0.1% (v/v) heat-inactivated FBS with optimally titrated concentrations of the antibodies CD3-AF700 (BioLegend, San Diego, CA, USA—UCHT1), CD14 -APC-Cy7 (BioLegend—HCD14), CD16-BV785 (BioLegend−3G8), CD19-APC-Cy7 (BioLegend—HIB19), CD56-BUV395 (BD Biosciences, San Jose, CA, USA—NCAM16.2), CD57-PEDazzle594 (BioLegend—HNK-1), KIR2DL1/KIR2DS5-PE (R&D Systems, MN, USA−143211), KIR2DL2/S2/L3-BV650 (BD Biosciences—DX27), KIR3DL1-BV421 (BioLegend—DX9), NKG2A-PECy7 (Beckman Coulter, CA, USA—Z199), and LIVE/DEAD fixable near-IR dye (Thermo Fisher Scientific) for 20 min at 4°C in the dark. Samples were subsequently washed in PBS then fixed with 0.5% (w/w) PFA (Sigma-Aldrich, MO, USA) before acquisition on a BD LSRFortessa flow cytometer.

### Uptake Assays and Mitochondrial Staining

2-NBDG and BODIPY FL C_16_ uptake assays were performed as previously described (21). Briefly, PBMCs were incubated in glucose-free RPMI (Thermo Fisher Scientific) supplemented with 50 μM 2-NBDG (Biomol, Hamburg, Germany), PBS supplemented with 12.5 μM BODIPY FL C_16_ (Thermo Fisher Scientific) or RPMI containing 10% (v/v) FBS supplemented with 100 nM MitoTracker Green and 12.5 nM MitoTracker Deep Red (Thermo Fisher Scientific) for 30 min at 37°C, 5% (v/v) CO_2_. Subsequently, surface staining was carried out as above. Cells were washed with PBS, fixed with 0.5% (w/w) PFA and then acquired on a BD LSRFortessa flow cytometer (BD Biosciences).

### NK Cell Degranulation Assay

Frozen PBMCs were defrosted and rested overnight with 1 ng/mL IL-15 (PeproTech, NJ, USA) then stimulated at an E:T ratio of 5:1 with K562 cells for 4 h in the presence of CD107a-BV510 antibody (BioLegend–H4A3) at 37°C, 5% (v/v) CO_2_, as previously described (22). Samples were subsequently washed in preparation for uptake assays, mitochondrial staining or antibody labeling for flow cytometry.

### Glycolysis and Oxidative Phosphorylation Inhibition Assays

PBMCs were incubated in glucose-free medium (Thermo Fisher Scientific) with 1.6−40 mM 2-deoxyglucose (Biomol) or in RPMI containing 10% (v/v) FBS with 1.6−40 μM of oligomycin (Biomol, Hamburg, Germany) for 2 h at 37°C, 5% (v/v) CO_2_. Following this, cells were washed and an NK cell degranulation assay was run using media matching that used during inhibition.

### Fluorescent Microscopy and Calcium Flux Assay

NK cells were enriched from freshly isolated PBMCs using an EasySep Human NK Cell Enrichment Kit (Stemcell, Vancouver, Canada) as per the manufacturer's guidelines and sorted using a BD FACS Aria-Fusion. NK cells were labeled with CD3-AF700, CD14-APC-Cy7, CD19-APC-Cy7, NKG2A-APC (Miltenyi Biotec, Bergisch Gladbach, Germany–REA110), LIVE/DEAD fixable near-IR dye and a combination of KIR2DL1/KIR2DS5-PE, KIR2DL2/S2/L3-PE (BioLegend–DX27), and KIR3DL1-PE (BioLegend–DX9) depending on specific donor genotype for 20 min at 4°C in the dark. Cells were sorted to select for NKG2A-educated NK cells and uneducated NK cells while excluding KIR-educated NK cells and were subsequently incubated overnight in RPMI containing 10% (v/v) FBS with 1 ng/mL IL-15. NK cells were washed then resuspended in calcium buffer (HBSS + 1 mM CaCl2 + 0.5 mM MgCl2 + 10 mM HEPES + 0.1% FBS) and stained with 4 μM Fura2 (Life Technologies, CA, USA) for 30 min at 37°C. Cells were resuspended in calcium buffer then observed under a fluorescent microscope. Biotinylated anti-NKp46 (BioLegend–9E2) and anti-2B4 (BioLegend–C1.7) antibodies were added after 3 min then streptavidin (BioLegend) after a further 2 min. Thapsigargin (Merck Millipore, MA, USA) was added at 17 min. The 340/380 ratio of Fura2 was recorded to measure intracellular calcium release by cells over time at 37°C using an incubator chamber.

### Software and Statistical Analysis

GraphPad Prism 8 (GraphPad Software, La Jolla, CA, USA) was used for statistical analyses and graphical display of the data. FlowJo 10 (FlowJo, LLC, Ashland, OR, USA) was used for analysis of flow cytometry data. Volocity 6.2.1 (PerkinElmer) was used for calcium signaling data acquisition and Fiji (23) was used for post-processing. FACSDiva 8 (BD) was used for flow cytometry data acquisition. For single comparisons the Wilcoxon matched-pairs signed rank test was used. For matched multiple comparisons, Friedman's test with Dunn's multiple comparisons test was used—otherwise Kruskal-Wallis test was used. Two-way ANOVA with Tukey's multiple comparisons was used for blockade experiments. *P*-values were multiplicity adjusted and adjusted *p*-values below 0.05 were considered statistically significant. All statistically significant differences are marked in figures by an asterisk and non-significant differences are unmarked.

### Data Availability

The datasets generated and analyzed during the current study are stored on an internal server at the Heinrich Pette Institute and are available from the corresponding author on reasonable request.

## Results

### Increased 2-NBDG and BODIPY Uptake in Educated vs. Uneducated NK Cells

**Figure 1:**
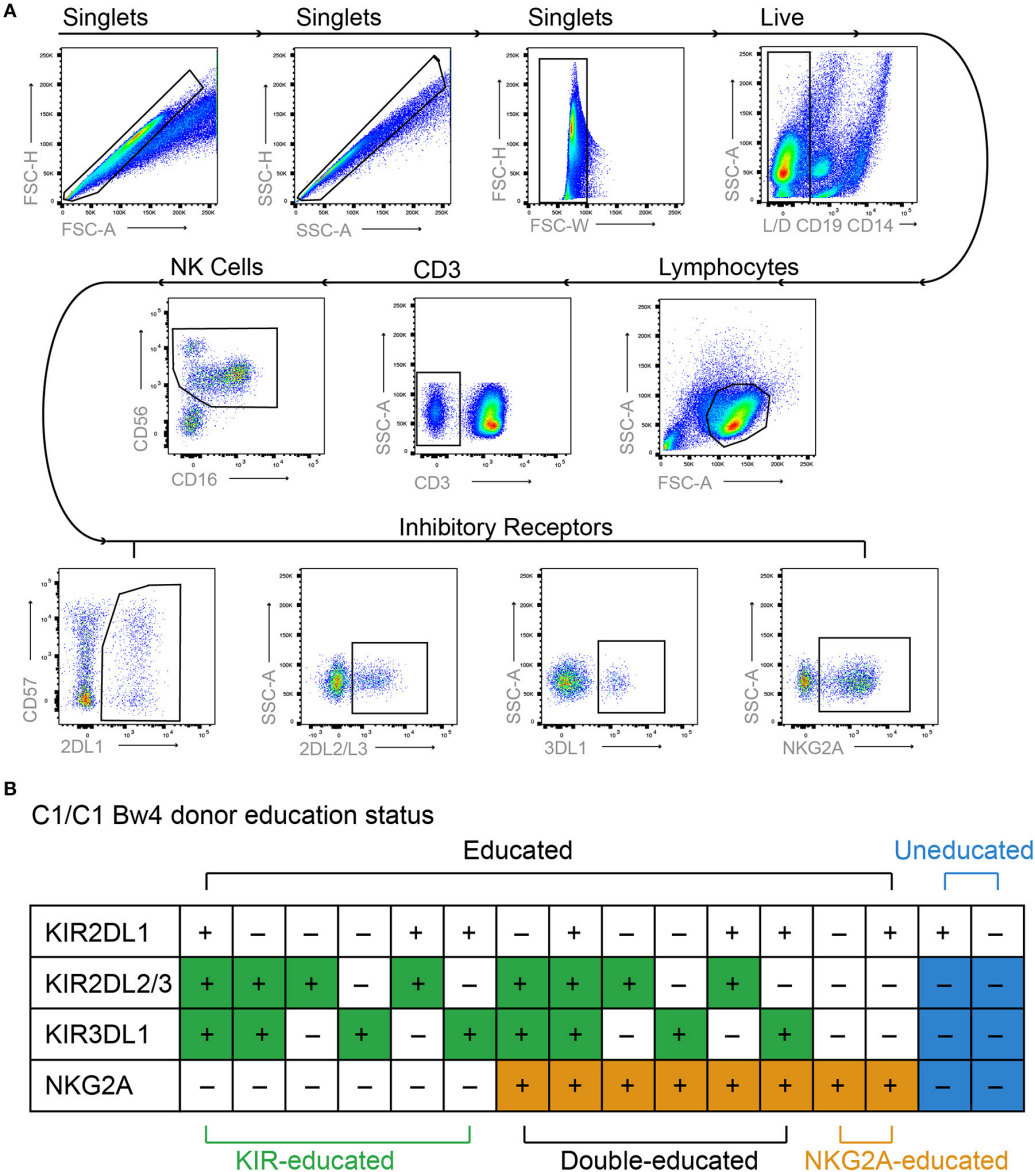
Gating strategy and NK cell education status. PBMCs were labeled with lymphocyte lineage markers as well as KIR2DL1, KIR2DL2/L3, KIR3DL1 NKG2A, and Live/Dead dye. **(A)** Doublets, dead cells, and non-NK cell lymphocytes were excluded from analysis and NK cells were identified based on expression of CD56 and CD16. Boolean gating was used to identify NK cells expressing different combinations of inhibitory receptors. **(B)** Educated and uneducated NK cells were identified based on inhibitory receptor expression and specific donor HLA genotype as exemplified for a donor homozygous for HLA-C1 and expressing the Bw4 epitope.

**Figure 2:**
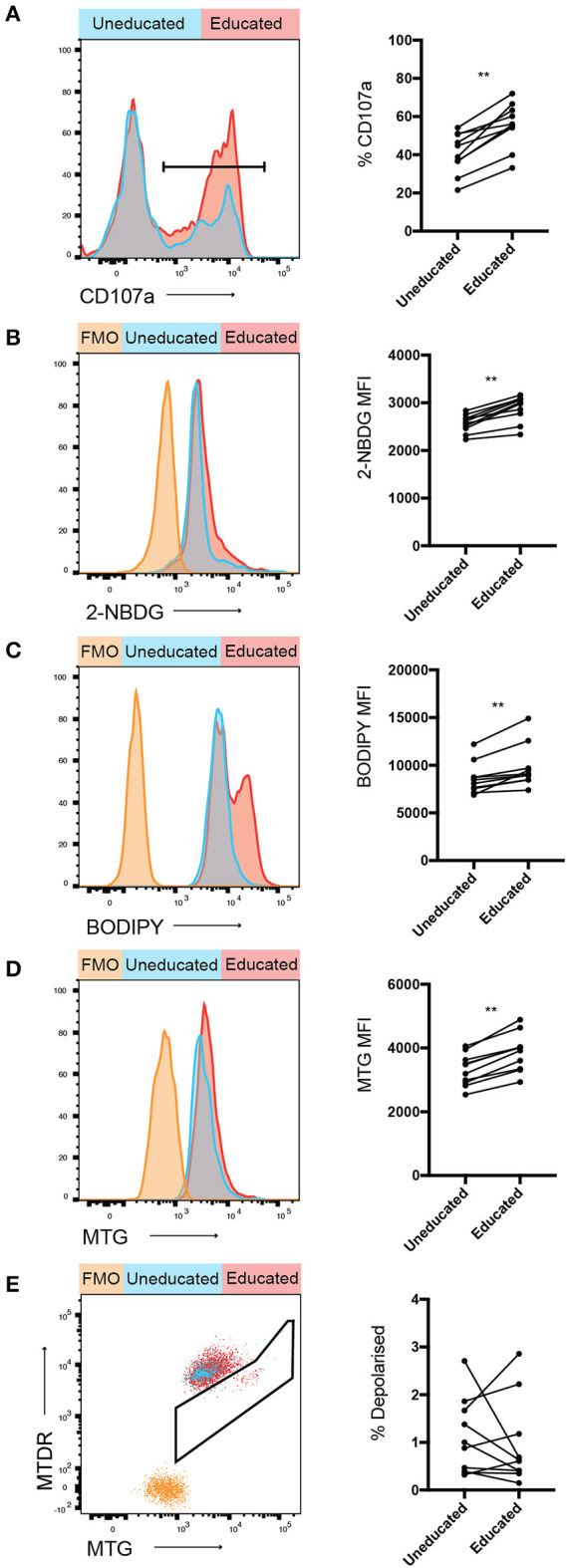
Increased 2-NBDG and BODIPY uptake in educated vs. uneducated NK cells. PBMCs were stimulated with K562 cells for 4 h in media containing anti-CD107a antibody. **(A)** Degranulation of uneducated and educated NK cell populations was determined. Immediately following stimulation, PBMCs were cultured in media containing **(B)** 2-NBDG; or **(C)** BODIPY and uptake of these metabolite analogs was assessed using flow cytometry. **(D)** Mitochondrial mass of stimulated educated and uneducated NK cells was determined using MitoTracker Green. **(E)** PBMCs were stained with MitoTracker DeepRed and the combination of MitoTracker Green and MitoTracker DeepRed was used to determine the mitochondrial polarization state of educated and uneducated NK cells. Left panels show representative histograms, right panels show pooled data from healthy donors. Data are representative of three independent experiments, mean ± *SD* shown, *n* = 10, **p < 0.01.

NK cells are educated during development through the engagement of inhibitory NK cell receptors with their ligands, and several studies have shown that the functional capacity of educated and uneducated NK cells differs (2, 3, 24–26). To investigate metabolic function, educated and uneducated NK cell populations were identified within bulk PBMCs from KIR/HLA genotyped healthy donors using flow cytometry to determine specific KIR and NKG2A expression (Figure 1A). Educated NK cells were defined as NK cells that expressed KIR2DL1, KIR2DL2/3, and/or KIR3DL1 in the presence of their respective ligands (HLA-C group 2, HLA-C group 1, HLA-Bw4) or NKG2A, as exemplified for one donor in Figure 1B. NK cells expressing both KIRs with their respective HLA ligands (educating KIRs) and KIRs that lacked their respective HLA ligands (non-educating KIRs) were also defined as educated, as our analysis demonstrated equivalent function with their inclusion (Supplementary Figure 1A). For the initial analyses, all KIR- and NKG2A-educated NK cells were pooled and their function was compared to uneducated NK cells, using degranulation (CD107a expression) following stimulation with K562 cells as a read out. In line with previous studies (16, 22), educated NK cells had significantly higher CD107a expression compared to uneducated NK cells, indicating an increased ability to respond to stimulation (Figure 2A).

Glucose, amino acids and fatty acids are critical metabolites used by cells to fuel glycolysis and oxidative phosphorylation. The usage of specific substrates further modulates immune cell responses (27). Uptake assays were performed to determine whether any of these metabolites were preferentially taken-up by educated or uneducated NK cells following activation. 2-NBDG, a fluorescent glucose analog, was used to assess uptake of glucose in NK cells following stimulation with K562 cells (Figure 2B). 2-NBDG uptake was significantly increased in educated compared to uneducated NK cells following stimulation with K562 cells, indicating an increased glucose demand. BODIPY FL C_16_ (BODIPY), a fluorescent palmitate, enabled the detection of fatty acid uptake in stimulated NK cells. Similar to 2-NBDG, BODIPY uptake was also significantly increased in educated compared to uneducated NK cells (Figure 2C). Taken together, these results demonstrate that educated NK cells have increased ability to uptake energy substrates.

Mitochondria are organelles critically involved in cellular energy production (28, 29). The membrane potential of mitochondria is important for effective oxidative phosphorylation, whereas membrane depolarisation impairs mitochondrial function. In order to determine the polarization state of mitochondria in educated compared to uneducated NK cells, cells were stained with MitoTracker Green (MTG; identifies all mitochondria) and MitoTracker Deep Red (MTDR; identifies polarized mitochondria), enabling an assessment of the overall polarization state of mitochondria within an NK cell. A significant difference was observed in the relative mitochondrial mass between uneducated and educated NK cells (Figure 2D), with educated cells having an enhanced mitochondrial mass, potentially allowing enhanced energy production. The frequency of depolarised mitochondria in uneducated compared to educated NK cells did not significantly differ (Figure 2E), indicating that the majority of educated and uneducated NK cells remained polarized with healthy mitochondrial function. Taken together, these results demonstrate clear metabolic differences between educated and uneducated NK cells. The enhanced energy substrate uptake, as well as mitochondrial mass, may contribute to generating energy for the increased function observed in educated NK cells.

### NKG2A-Educated NK Cells Have Increased 2-NBDG Uptake and BODIPY Uptake Compared to Uneducated or KIR-Educated NK Cells

**Figure 3:**
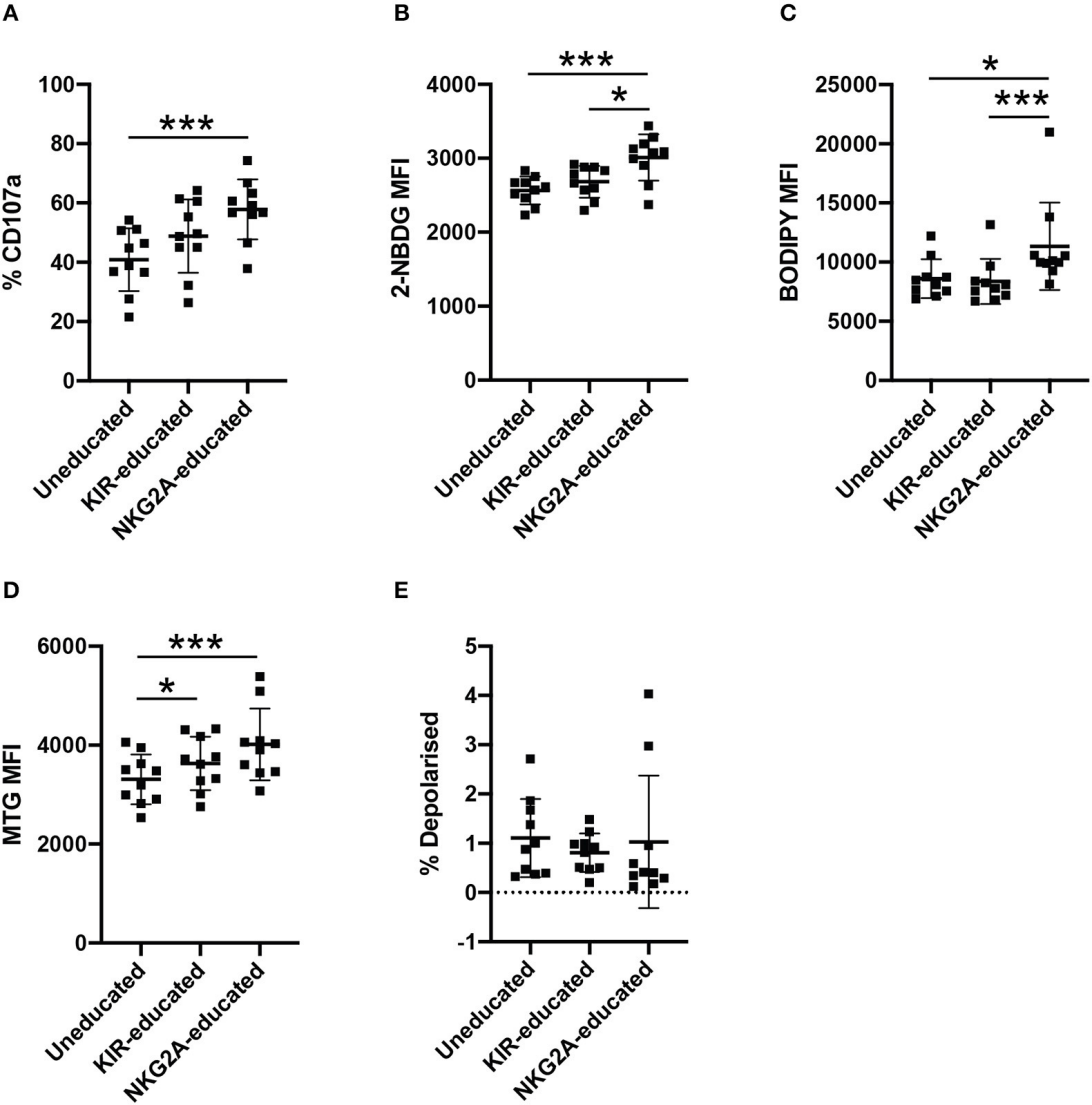
NKG2A-educated NK cells have increased 2-NBDG and BODIPY uptake compared to uneducated or KIR-educated NK cells. PBMCs were stimulated with K562 cells for 4 h in media containing anti-CD107a antibody. **(A)** Degranulation of uneducated NK cells or those educated *via* KIR or NKG2A was determined in bulk NK cells. Immediately following stimulation, PBMCs were cultured in media containing **(B)** 2-NBDG; or **(C)** BODIPY and uptake of these metabolite analogs was assessed in uneducated NK cells or those educated *via* KIR or NKG2A. **(D)** Mitochondrial mass of uneducated NK cells or those educated *via* KIR or NKG2A was determined using MitoTracker Green. **(E)** Stimulated PBMCs were stained with MitoTracker Green and MitoTracker DeepRed to determine the polarization state of uneducated NK cells or those educated *via* KIR or NKG2A. Data are representative of three independent experiments, mean ± *SD* shown, *n* = 10, *p < 0.05, ***p < 0.001.

Inhibitory receptors are differentially expressed between NK cells, resulting in NK cell populations educated by different inhibitory receptor repertoires. The majority of inhibitory KIRs and NKG2A have two intracytoplasmic immunoreceptor tyrosine-based inhibition motifs (ITIMs) but ITIMs are arranged in the reverse orientation in KIRs compared to NKG2A, suggesting they may have differing capacities to propagate inhibition (30, 31). Considering the possible differences between NK cells educated *via* KIRs or NKG2A, metabolite uptake was further compared between KIR- and NKG2A-educated NK cells. CD107a expression following stimulation with K562 cells was higher in both educated populations compared to uneducated NK cells and highest in NKG2A-educated NK cells (Figure 3A). 2-NBDG uptake was significantly enhanced in the NKG2A-educated compared to both KIR-educated NK cells and uneducated NK cells (Figure 3B). BODIPY uptake was also significantly increased in NKG2A-educated NK cell populations compared to both uneducated and KIR-educated NK cells (Figure 3C). These results suggest that differences in metabolite uptake between educated and uneducated NK cells are driven by NK cells educated *via* NKG2A.

CD56^bright^ and CD56^dim^ NK cells differ in the expression of NKG2A, and it has been suggested that CD56^bright^ NKG2A^+^ NK cells represent more immature NK cell populations (32). CD57, on the other hand, indicates a more mature differentiation state in NK cells, however has also been linked to lymphocyte exhaustion (32). To determine whether the observed differences in metabolite uptake were due to differences in NK cell maturity rather than education, NK cells were separated into CD56^bright^ and CD56^dim^ populations for further analysis (Supplementary Figure 2). CD56^dim^ NKG2A-educated and KIR-educated NK cells had increased CD107a expression compared to CD56^dim^ uneducated NK cells (Supplementary Figure 2A). Within the CD56^dim^ NK cell population, NKG2A-educated NK cells exhibited the highest 2-NBDG uptake, and 2-NBDG uptake was significantly increased compared to uneducated NK cells. 2-NBDG uptake was more variable within the CD56^bright^ NK cells, where no differences were observed in 2-NBDG uptake between groups (Supplementary Figure 2B). Interestingly, 2-NBDG uptake was significantly increased in both CD57^+^ and CD57^−^ NKG2A-educated NK cells compared to uneducated NK cells (Supplementary Figure 3B). Uptake of BODIPY was significantly higher in all CD56^bright^ NK cell populations compared to CD56^dim^ NK cells (Supplementary Figure 2C, *p* < 0.0001). BODIPY uptake was also significantly increased in NKG2A-educated NK cells compared to KIR-educated and uneducated NK cells within CD56^dim^ populations as well as the CD57^−^ and CD57^+^ subsets (Supplementary Figures 2C, 3C). The relative mitochondrial mass in bulk and CD56^dim^ educated NK cells was significantly increased compared to uneducated NK cells but the difference was more pronounced in NKG2A-educated NK cells than KIR-educated NK cells (Figure 3D, Supplementary Figure 2D). The same difference was also observed in CD57^+^ NK cells while, in contrast, CD57^−^ NKG2A-educated NK cells had significantly increased mitochondrial mass compared to both KIR-educated and uneducated NK cells (Supplementary Figure 3D). This increased mitochondrial mass is suggestive of increased metabolic activity, corroborating the increased uptake of both 2-NBDG and BODIPY by the NKG2A-educated NK cell population. Mitochondria in all NK cell subsets were largely polarized, though CD57^+^ NKG2A-educated NK cells had a significantly lower frequency of depolarised mitochondria compared to uneducated NK cells (Figure 3E, Supplementary Figures 2E, 3E). Together, these results show that the NKG2A-educated subset of NK cells exhibit superior metabolic activity compared to uneducated NK cells and KIR-educated NK cells. CD57^+^ NK cells that have not undergone education had decreased metabolic activity and functionality (as measured by CD107) as well as unhealthy, depolarised mitochondria, indicating impaired responses. CD56^dim^CD57^−^ NK cells retained significant differences between NKG2A-educated and uneducated NK cells when compared to the bulk CD57^−^ population, indicating that enrichment for CD56^bright^ cells within the NKG2A-educated population did not dictate the differences observed (Supplementary Figures 2, 3).

### NKG2A-Educated NK Cells Are More Resilient to Inhibition of Oxidative Phosphorylation

**Figure 4:**
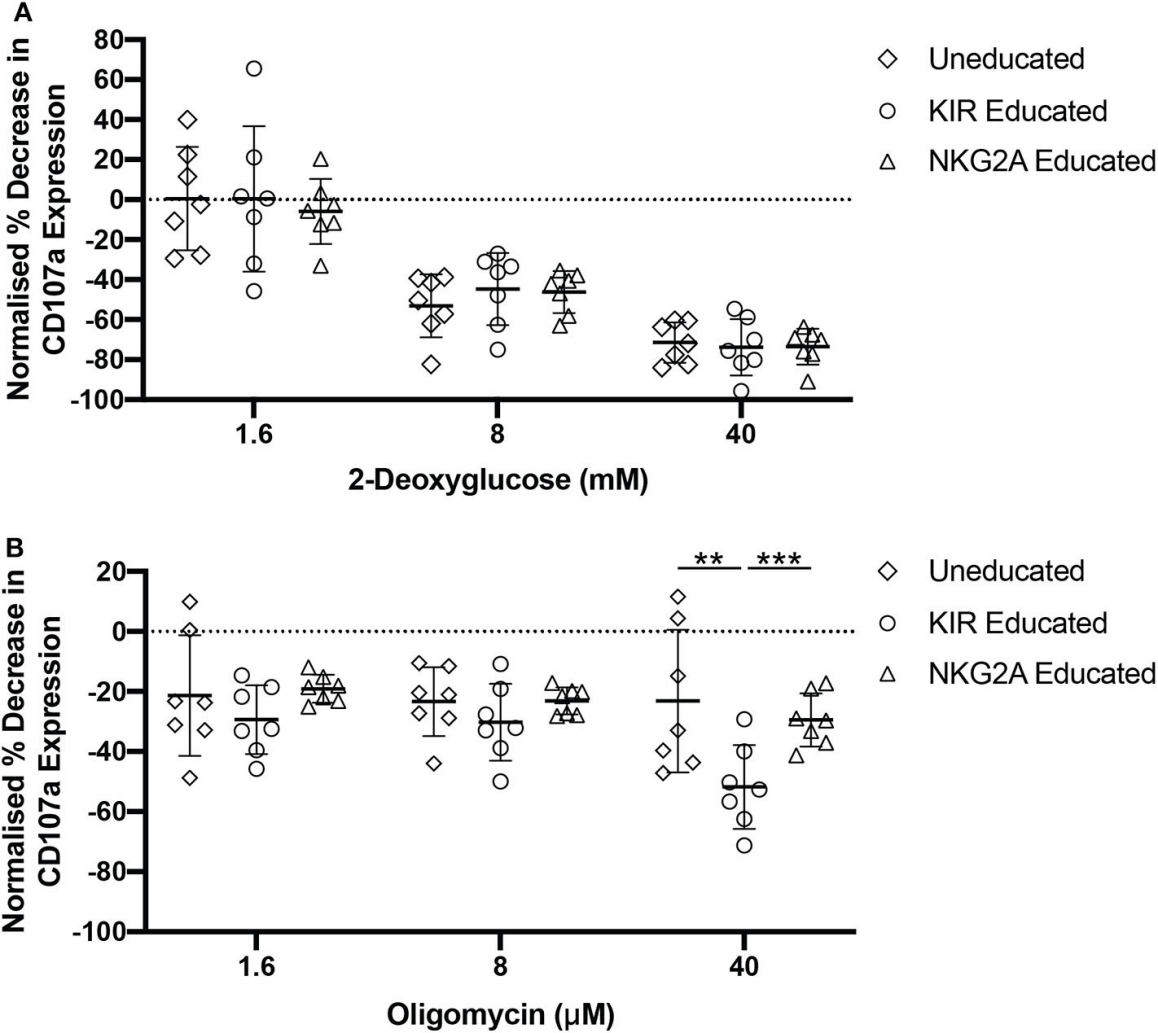
NKG2A-educated NK cells are more resilient to oligomycin pre-treatment. PBMCs were incubated in media containing 2-DG, oligomycin, or both and subsequently stimulated for 4 h with K562 cells. Degranulation was assessed in uneducated NK cells or those educated *via* KIRs or NKG2A following inhibition with **(A)** 2-DG; or **(B)** oligomycin. Data are representative of three independent experiments, mean ± *SD* shown, *n* = 7, **p < 0.01, ***p < 0.001.

Oxidative phosphorylation and glycolysis are both important processes for the production of ATP. Aerobic glycolysis does not fuel oxidative phosphorylation but is an important pathway for immune cells as it provides a fast source of ATP and molecules required for cellular proliferation. Oxidative phosphorylation and glycolysis can both be blocked using chemical inhibitors. Oligomycin blocks the proton channel of ATP synthase, thus preventing any ATP production *via* oxidative phosphorylation, while 2-deoxyglucose (2-DG) is an altered glucose molecule that functions through competitive inhibition of the glycolysis pathway. As we observed that both 2-NBDG- and BODIPY-uptake were increased in NKG2A-educated compared to KIR-educated NK cells, glycolysis or oxidative phosphorylation were blocked to determine whether either of these pathways was more functionally relevant in these NK cell populations. NK cells were pre-treated with either oligomycin or 2-DG before quantification of CD107a-expression following stimulation with K562 cells. Pre-treatment with 2-DG resulted in similar relative CD107a expression decrease in educated and uneducated NK cells (Figure 4A). Pre-treatment with high concentrations of oligomycin resulted in a decrease in CD107a expression in both KIR- and NKG2A-educated NK cells although KIR-educated NK cells were more strongly affected (Figure 4B). Blockade of both pathways almost completely inhibited CD107a expression by both NK cell subsets (data not shown). These results indicate that glycolysis is of equal importance for degranulation in response to K562 cells in both educated and uneducated NK cells. Interestingly, NKG2A-educated NK cell degranulation was found to be more resilient to blockade of oxidative phosphorylation than KIR-educated NK cells, possibly due to the increased mitochondrial mass and fatty acid uptake previously observed for NKG2A-educated NK cells (Figures 3C,D).

### Educated NK Cells Have Increased Peak Calcium Signal Compared to Uneducated NK Cells Following Stimulation

**Figure 5:**
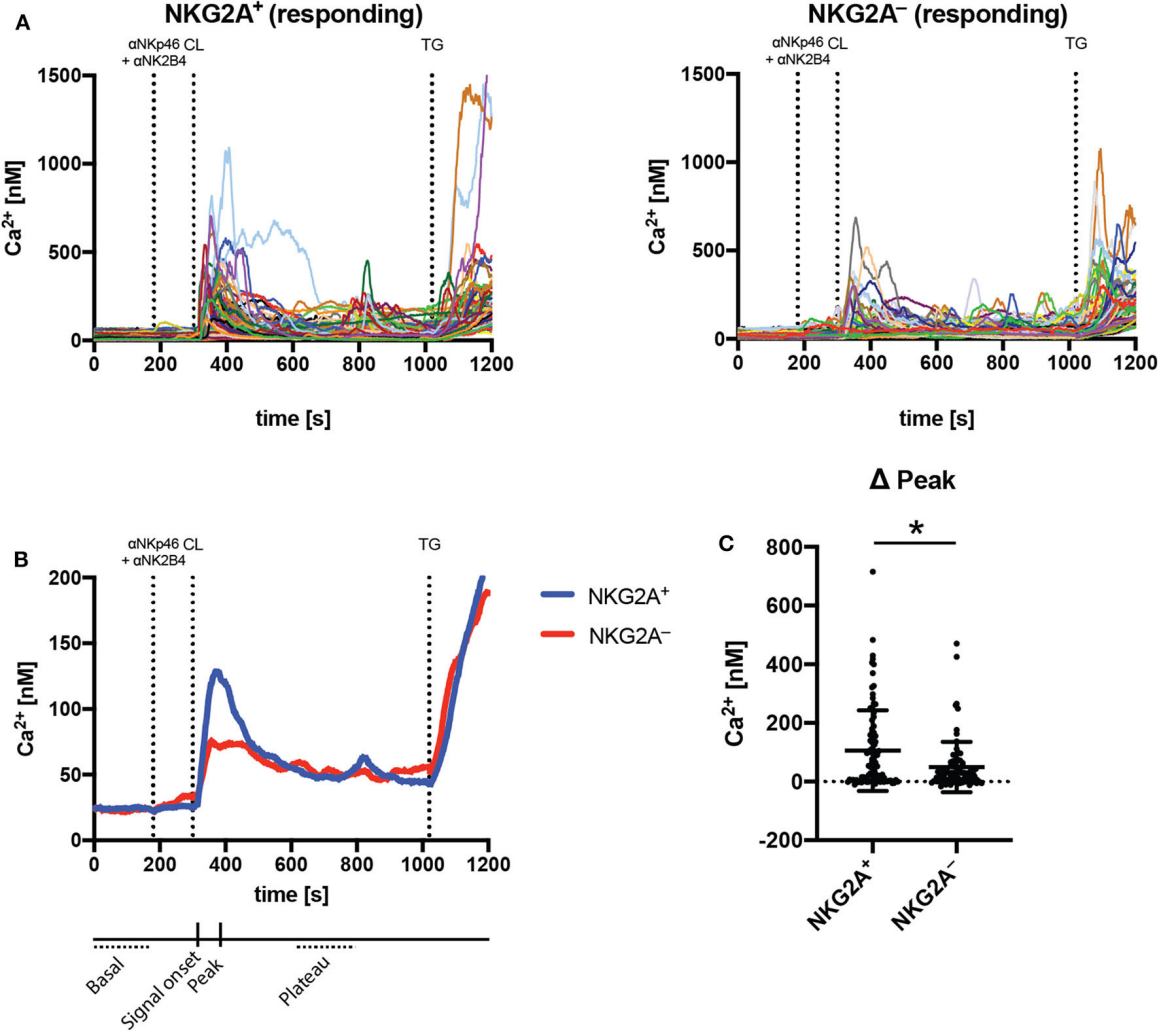
NKG2A-educated NK cells have increased peak calcium signal compared to uneducated NK cells following stimulation. Calcium signaling was assessed using fluorescent microscopy and the ratiometric dye Fura2 in sorted NKG2A^+^ and NKG2A^−^ NK cell populations. **(A)** Intracellular calcium was detected following stimulation with cross-linked anti-NKp46 and anti-2B4 antibodies in individual responding NK cells over time; **(B)** the mean calcium signal of NKG2A^+^ and NKG2A^−^ NK cells over time; and **(C)** the peak signal following cross-linking. Data are from seven pooled experiments, mean ± *SD* shown, *n* = 7, *p < 0.05.

Calcium signaling is required for effector function of NK cells, and it has been recently shown that secretory lysosomes storing calcium may contribute to functional differences between educated and uneducated NK cells (9). mTORC1 is activated by lysosomal components (33), suggesting a link between NK cell education, metabolism and calcium signaling. To determine whether calcium signaling was increased in educated compared to uneducated NK cells, NKG2A-educated and uneducated NK cells were sorted, loaded with Fura-2 calcium indicator and then stimulated by cross-linking anti-NKp46 and anti-2B4 antibodies. Calcium signaling was analyzed over time using fluorescence microscope-based calcium imaging. An initial calcium peak was observed in both NKG2A^+^ and NKG2A^−^ NK cells responding to the cross-linking of stimulating antibodies with streptavidin (Figures 5A,B). A similar frequency of NK cells responding to stimulation was observed in both NKG2A^+^ and NKG2A^−^ populations and the basal calcium concentration between these populations was not different (Supplementary Figures 4A,B). Calcium signaling was similar between NKG2A^+^ and NKG2A^−^ cells 25 s following cross-linking but the peak signal of NKG2A^+^ NK cells was significantly higher than NKG2A^−^ NK cells (Figures 5B,C, Supplementary Figure 4C). Following this initial difference, the calcium signal remained similar between populations, including after stimulation with SERCA inhibitor thapsigargin as positive control (Figure 5B, Supplementary Figure 4D). These results demonstrate that peak calcium concentration is more pronounced in NKG2A-educated NK cells as compared to those not educated *via* NKG2A, in line with the increased metabolic activity and degranulation in response to K562 cells observed in NKG2A^+^ NK cells.

## Discussion

Cellular metabolism is critical for the function of immune cells. We investigated metabolic differences between educated and uneducated NK cells and observed that educated NK cells had elevated uptake of nutrients compared to uneducated NK cells, indicating increased metabolic activity of educated NK cells. Further characterization of educated NK cell subsets revealed that NK cells educated through NKG2A exhibit superior metabolic activity, and blockade of metabolic pathways demonstrated that NKG2A^+^ NK cells were more resilient to reduction in oxidative phosphorylation compared to KIR^+^ NK cells. In line with these metabolic functions, calcium signaling within the NKG2A^+^ population of NK cells was also increased compared to uneducated NK cells. Collectively, these data show that educated NK cells, and in particular those NK cell populations educated through NKG2A, are more metabolically active than uneducated NK cells, allowing for enhanced functional activity.

Glucose and fatty acids are both important metabolites for energy production. Previous work has demonstrated differences in 2-NBDG uptake between CD56^bright^ and CD56^dim^ human NK cell populations, and implicated glucose as an important metabolite for fuelling both glycolysis and oxidative phosphorylation (13). These observations are supported by results in mice, where both oxidative phosphorylation and glycolysis were upregulated in NK cells following cytokine stimulation in a glucose-dependant manner (14, 34). Comparing educated to uneducated NK cells, we observed increased 2-NBDG uptake in educated NK cells compared to uneducated NK cells, but a much larger difference in the uptake of BODIPY. This difference was particularly pronounced in NKG2A-educated NK cells. Recent work by Michelet et al. (35) has shown that fatty acid-accumulation in the NK cells of obese patients reduces their cytotoxic capacity *via* inhibition of glycolytic machinery in both the setting of cytokine-simulated activation and in an *in vivo* mouse model of melanoma. It was shown here that the more functional NKG2A-educated NK cell population took up increased levels of fatty acid compared to uneducated NK cells. Obesity is associated with exhaustion of lymphocyte populations and it is possible that in the setting of obesity, this subset would preferentially become dysfunctional compared to KIR-educated NK cells due to increased fatty acid accumulation. Although it is possible that fatty acids act as inhibitory molecules for NK cells under some conditions, the increased demand observed in the present work by highly functional NK cells suggests a more beneficial effect promoting NK cell function.

Although fatty acids are important for oxidative phosphorylation in T lymphocytes, the same has not been demonstrated in NK cells. We observed that BODIPY uptake was significantly higher in NKG2A-educated NK cells compared to either KIR-educated or uneducated NK cells. Interestingly, fatty acids have been shown to be a major source of acetyl-CoA for acetylation of histones in hepatocytes, which can lead to specific fatty-acid-driven gene expression patterns (36). These data suggest that increased uptake and utilization of fatty acid may lead to an altered gene expression profile in NKG2A-educated NK cells, which could further explain the enhanced metabolic function in this subset. Certain short-chain fatty acids (SCFA) have also been shown to act as histone deacetylase (HDAC) inhibitors, which are being investigated as potential anti-cancer treatments (37, 38). In the context of cancer treated with SCFA HDAC inhibitors, NKG2A-educated NK cells may be able to retain increased functionality through fatty acid oxidation of the same inhibitor. Indeed, the SCFA butyrate has been shown to enhance oxidative phosphorylation in T cells and has shown potential as a colorectal cancer treatment (39, 40). Collectively, these data suggest that NKG2A-educated NK cells could be an effective cancer co-therapy alongside SCFA HDAC inhibitors.

Oligomycin is a widely used inhibitor of ATP synthase and thus of ATP production *via* oxidative phosphorylation. It has previously been demonstrated that inhibition of oxidative phosphorylation with oligomycin in NK cells reduces their cytotoxicity and cytokine production (17). We observed that NKG2A-educated NK cell populations were more resistant to inhibition of oxidative phosphorylation than KIR-educated NK cells. Interestingly, work by Schafer et al. demonstrated an increased susceptibility of uneducated NK cells to oligomycin treatment but not of KIR-educated NK cells (17). This observed difference may be due to the use of sorted and expanded NK cells by Schafer et al. or due to their use of 721.221 cells for stimulation compared to K562s in this study. The increased mitochondrial mass in NKG2A-educated NK cells may contribute to the decreased sensitivity to blockade of oxidative phosphorylation. It is possible that NKG2A-educated NK cells are able to more effectively utilize glycolysis and are thus less reliant on ATP generated through oxidative phosphorylation. Inhibition of glycolysis alone with 2-DG similarly decreased the relative cytotoxicity of NKG2A- and KIR-educated NK cells. Together, this suggests a greater flexibility in energy production by NKG2A-educated NK cells, as blockade of oxidative phosphorylation alone had less impact on their functionality compared to KIR-educated NK cells. Interestingly, combined blockade of both oxidative phosphorylation and glycolysis was required to completely abrogate degranulation of NKG2A-educated NK cells in response to K562 cells. These results suggest that metabolic drugs such as metformin and hydroxychloroquine, which are being investigated for their use as anti-cancer therapies, may allow NKG2A-educated NK cells to retain cytotoxic function while simultaneously inhibiting cancer cell function.

The interactions between NKG2A, KIRs and their respective HLA ligands have emerged in distinct periods during evolution, with NKG2A/HLA-E interactions pre-dating interactions between KIRs and HLA-ABC (26). Horowitz et al. (26) have shown that the −21 position in the HLA-B leader sequence can significantly affect the educating environment to favor an NKG2A-dominant or KIR-dominant NK cell response, depending on whether a methionine or threonine residue is present. Donors with a methionine at the −21 position of at least one HLA-B allele displayed a more functional NKG2A^+^ population than those with only threonine residues. These results demonstrate the importance during education of not only expression of an inhibitory receptor, but also the level of expression of the respective ligands. Donors with more strongly NKG2A-educated NK cell responses also expressed higher levels of HLA-E, and the same was true for KIR-educated NK cells and their respective HLA ligands (26). These data indicate that not only are differences in specific signaling pathways important for NK cell education but also the quantity or intensity of those signals. The majority of inhibitory receptors expressed on NK cells contain ITIMs to propagate signaling that includes the dephosphorylation of Vav1 and the phosphorylation of Crk (41). Interestingly, the ITIM domains of NKG2A and KIRs are arranged in opposite orientations, which may lead to differences in downstream inhibitory signaling despite the utilization of similar inhibitory mechanisms (30, 31). Differences in receptor and ligand expressions, and the strength of their interactions and downstream signaling, may therefore work together in modulating the metabolic activity of individual NK cell populations, resulting in the metabolic differences observed between NKG2A- and KIR-educated NK cells. In our study, fatty acid uptake was found to remain increased in NKG2A-educated NK cells in donors with or without a methionine at the −21 position of HLA-B (Supplementary Figure 5). Taken together, these results indicate that the increased BODIPY uptake seen in NKG2A-educated compared to KIR-educated NK cells is a robust change, though more investigation is required to fully determine whether −21 M or T on HLA-B impacts this difference.

Human cytomegalovirus (HCMV) is known to modulate the function of specific NK cell subsets. Of note, CD56^dim^CD57^+^NKG2C^+^ adaptive NK cells have been reported to have increased mitochondrial mass and increased mitochondrial membrane potential compared to canonical NK cells (42). CD57^+^NKG2A^+^ NK cells have also been demonstrated to have increased function in HCMV seropositive individuals (43). Here, we observed an increased uptake of fatty acid and 2-NBDG as well as increased mitochondrial mass in CD56^dim^CD57^+^NKG2A^+^ NK cells compared to uneducated NK cells but also in CD56^dim^CD57^−^NKG2A^+^ NK cells (Supplementary Figures 2B–D). No information on HCMV serostatus was available for the donors in this study and antibodies against NKG2C were not included in the analysis. We are therefore unable to assess the influence of HCMV on the increased metabolic function of the CD56^dim^CD57^+^NKG2A^+^ NK cell subset.

The cytotoxic activity of NK cells is dependent on several signaling events, including calcium signaling. NK cells lacking the calcium channel ORAI1 or its regulator protein STIM1 have been shown to have greatly reduced cytotoxic activity (10). Goodridge et al. have furthermore suggested that secretory lysosomes may play a role in the functional differences observed between educated and uneducated NK cells (9). In their work, GPN (glycyl-l-phenylalanine 2-naphthylamide) was used to induce lysosomal calcium release, which has been reported to act in a non-specific fashion (44, 45). Thus, although calcium signaling is important for NK cell function, additional studies are required to determine the role of lysosomal calcium stores in NK cell function. Here, we showed that NKG2A-educated NK cells had increased peak calcium signaling compared to uneducated NK cells following stimulation through NKp46 and 2B4. This, along with previous work, suggests an important role for calcium signaling in the functional differences between educated and uneducated NK cells. Interestingly, mTOR is known to be regulated through lysosomal interactions but also to regulate lysosomal function (46, 47). These data suggest an interplay between calcium signaling, metabolic signaling and NK cell education to determine NK cell function. Taken together, our results demonstrate that specific metabolic profiles correspond to functional differences between educated and uneducated NK cells, and indicate that metabolic modulation may be used to enhance NK cell functions, for example in the context of anti-cancer therapies.

## Data Availability Statement

The raw data supporting the conclusions of this article will be made available by the authors, upon reasonable request.

## Ethics Statement

The studies involving human participants were reviewed and approved by the ethical committee of Ärztekammer Hamburg, Germany (PV4780). The patients/participants provided their written informed consent to participate in this study.

## Author Contributions

AH, B-PD, CK, MB, and MA designed the experiments. AH, B-PD, FM, and GM carried out the experiments. AH, B-PD, and FM analyzed the data. AH wrote the manuscript. JS and AS provided resources. All authors reviewed the manuscript.

## Conflict of Interest

The authors declare that the research was conducted in the absence of any commercial or financial relationships that could be construed as a potential conflict of interest.
