# Supplementary material for: High Metabolic Function and Resilience of NKG2A-Educated NK Cells
Source: Front Immunol. 2020 Sep 30;11:559576. doi: 10.3389/fimmu.2020.559576 (PMC7554334; doi:10.3389/fimmu.2020.559576)
Supplement: Supplementary file 1 [file Data_Sheet_1.pdf]

High Metabolic Function and Resilience of NKG2A-educated NK Cells

Andrew J. Highton[1], Björn-Philipp Diercks[2], Franziska Möckl[2], Gloria Martrus[1], Jürgen Sauter[3], Alexander H. Schmidt[3], Madeleine J. Bunders[1], Christian Körner[1], Andreas H. Guse[2], Marcus Altfeld[1]*

[1]Research Department Virus Immunology, Heinrich Pette Institute, Leibniz Institute for Experimental Virology, Hamburg, Germany

[2]The Calcium Signalling Group, Department of Biochemistry and Molecular Cell Biology, University Medical Centre Hamburg-Eppendorf, Hamburg, Germany

[3]Deutsche Knochenmarkspenderdatei (DKMS), Tübingen, Germany

*** Correspondence:**

Marcus Altfeld

marcus.altfeld@leibniz-hpi.de

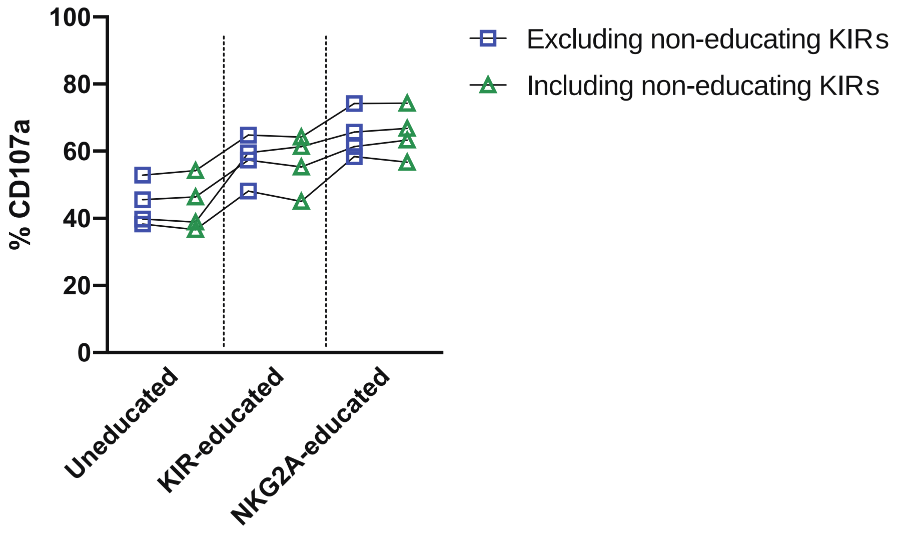

Supplementary Figure 1. Comparison of educated NK cells including or excluding non-educating KIRs.

PBMCs were stimulated with K562 cells for 4 hours in media containing anti-CD107a antibody. (A) Degranulation of uneducated NK cells or NK cells educated via KIRs or NKG2A was quantified. Open blue squares show NK cells from a gating strategy excluding NK cells expressing non-educating KIRs. Open green triangles show a gating strategy including NK cells expressing non-educating KIRs. Donors expressing HLA-C1, HLA-C2 and Bw4 in combination were excluded from analysis due to an unchanged gating strategy, n=4.

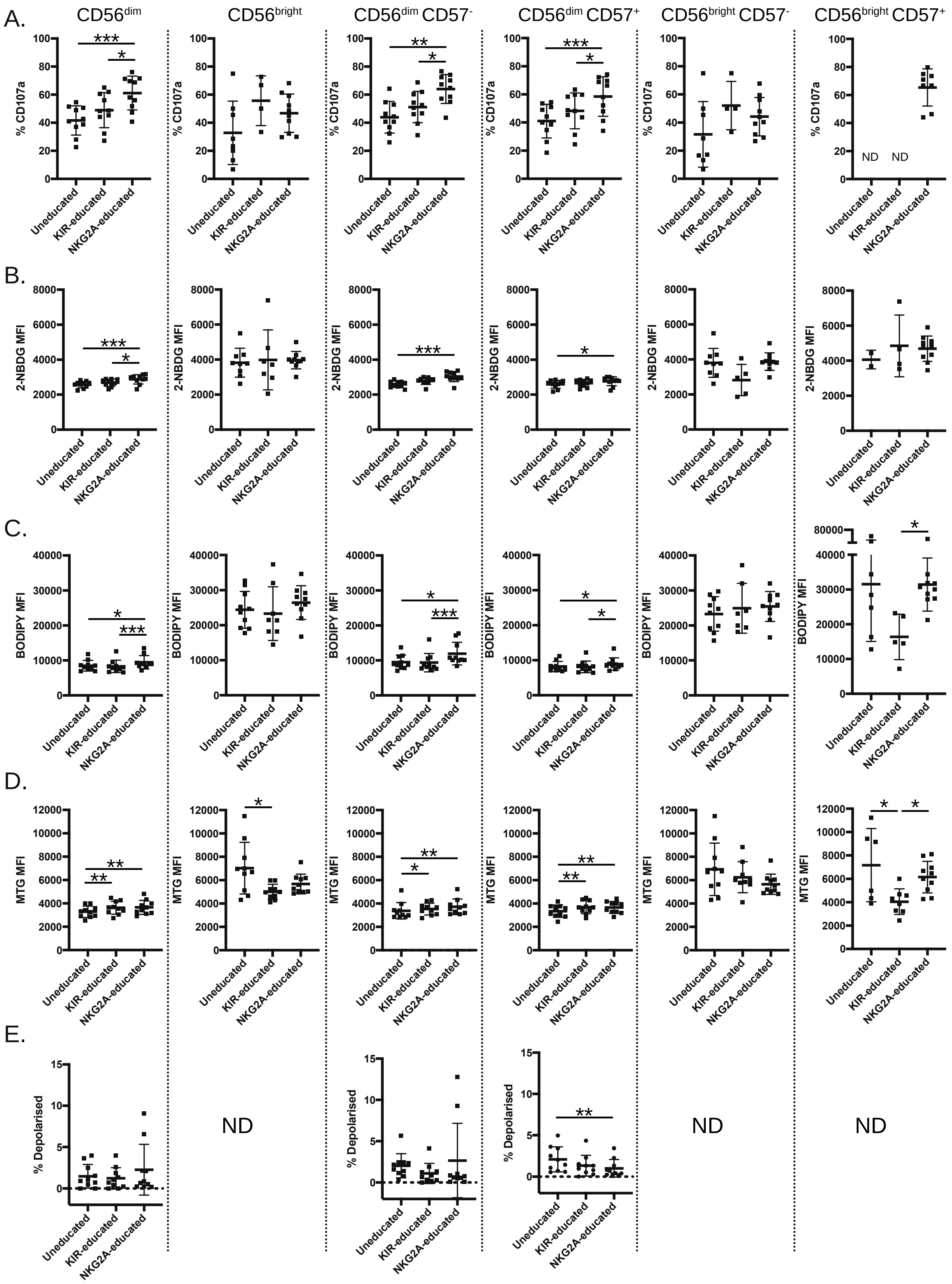

Supplementary Figure 2. **CD56$_{\mathrm{dim}}$ NKG2A-educated NK cells have increased 2-NBDG and BODIPY uptake compared to uneducated or KIR-educated NK cells.**

PBMCs were stimulated with K562 cells for 4 hours in media containing anti-CD107a antibody. (A) Degranulation of uneducated NK cells or those educated via KIR or NKG2A was determined in

CD56$_{bright/dim}$, CD56$_{dim}$CD57$_{-}$,CD56$_{dim}$CD57$_{+}$,CD56$_{bright}$CD57$_{-}$ and CD56$_{bright}$CD57$_{+}$ subsets. Immediately following stimulation, PBMCs were cultured in media containing (B) 2-NBDG; or (C) BODIPY FL C_{16} and uptake of these metabolite analogues was assessed in uneducated NK cells or those educated via KIRs or NKG2A. (D) Mitochondrial mass of uneducated NK cells or those educated via KIR or NKG2A was determined using MitoTracker Green. (E) Stimulated PBMCs were stained with MitoTracker Green and MitoTracker DeepRed to determine the polarisation state of uneducated NK cells or those educated via KIR or NKG2A. Due to low cell numbers in the CD56$_{bright}$ subset, some data points are excluded. Data are representative of three independent experiments, mean ± SD shown, n=10, * $p < 0.05$, ** $p < 0.01$, *** $p < 0.001$.

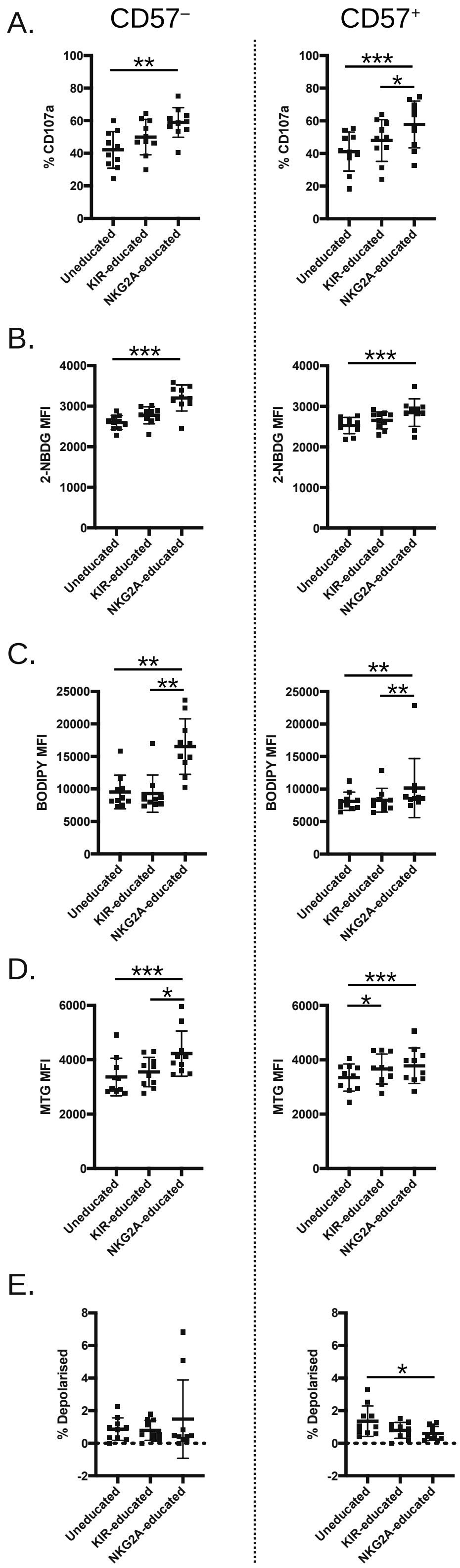

Supplementary Figure 3. **CD57+ and CD57- NKG2A-educated NK cells have increased 2-NBDG and BODIPY uptake compared to uneducated NK cells.**

PBMCs were stimulated with K562 cells for 4 hours in media containing anti-CD107a antibody. (A) Degranulation of uneducated NK cells or those educated via KIRs or NKG2A was determined in CD57-

and CD57+ subsets. Immediately following stimulation, PBMCs were cultured in media containing (B) 2-NBDG; or (C) BODIPY FL C_{16} and uptake of these metabolite analogues was assessed in uneducated NK cells or those educated via KIR or NKG2A. (D) Mitochondrial mass of uneducated NK cells or those educated via KIRs or NKG2A was determined using MitoTracker Green. (E) Stimulated PBMCs were stained with MitoTracker Green and MitoTracker DeepRed to determine the polarisation state of uneducated NK cells or those educated via KIRs or NKG2A. Data are representative of three independent experiments, mean ± SD shown, n=10, * $p < 0.05$, ** $p < 0.01$, *** $p <0.001$.

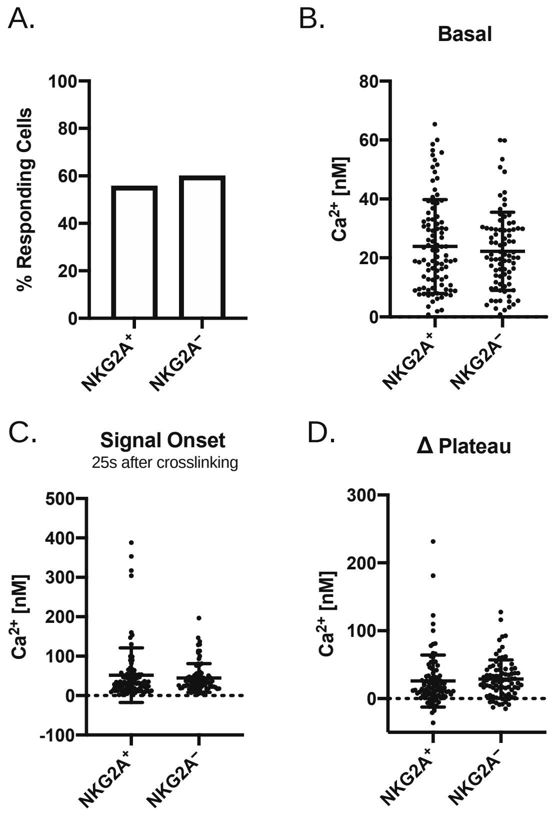

Supplementary Figure 4. **Basal and plateau calcium signals do not differ between NKG2A-educated and uneducated NK cells.**

Calcium signalling was assessed using fluorescent microscopy and the ratiometric dye Fura2 in sorted NKG2A+ and NKG2A- NK cell populations. (A) The percentage of total cells responding to stimulation with cross-linked anti-NKp46 and anti-2B4; (B) basal calcium signal between 0 – 200 seconds; (C) signal onset 25 seconds after cross-linking activating antibodies with streptavidin; and (D) the calcium signal plateau between 600 – 800 seconds, all depicted as calcium concentrations. Data are from 7 pooled experiments, mean ± SD shown, n=7.

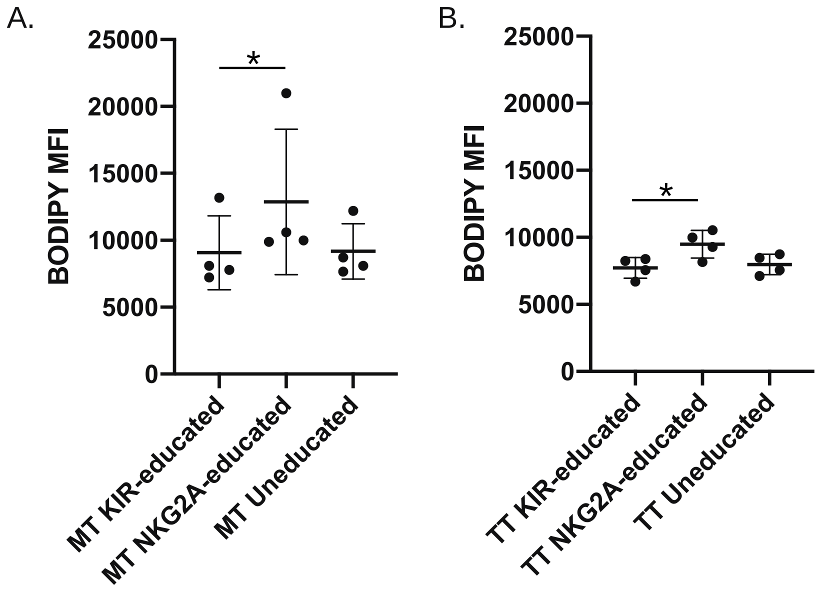

Supplementary Figure 5. NKG2A-educated NK cells have increased BODIPY uptake in donors with M/T or T/T at -21 HLA-B.

PBMCs were stimulated with K562 cells for 4 hours and subsequently incubated with BODIPY FL C_{16} to assess fatty acid uptake. Data represents donors with (A) a methionine and a threonine present at the -21 position on each HLA-B allele; or (B) only threonine residues. Data are representative of two independent experiments, mean ± SD shown, n=4, * $p < 0.05$.
